# 

**Published:** 1921-08-13

**Authors:** 


August 13 1921] [Price Twopence

### The Hospital

### The Workers' Newspaper of Administrative Medicine and Institutional Life, Administration, National Insurance and Health.

CONTENTS

Co-ordination in Local Units... ... ... ... 323
Publicity and Public Health... ... ... ... 323
The Red Cross Report... ... ... ... ... 324
A Mass of Interesting Detail.
Women in Medicine ... ... ... ... ... 324
Notes of the Week ... ... ... ... ... 325
Plans and Prospects at Cardiff—Regional Committees and New Hospitals—A Misunderstanding at Brighton—Annual Meetings Away from Home—The Doctors and St. Luke's Hospital—No Paying Patients at Camberwell Infirmary—The School-Teacher's Opportunity—Foresters and Panel Doctors—Pensions for Seamen—The Miners' Halfpenny at Worksop.
Alimentary Disorders of Infants ... ... 327
How Do They Really Arise?
Transplantation of Eyes ... ... ... ... 328
The Oxford Plan for Devonshire? ... ... ... 328
The Problem of the Insane ... ... ... ... 329
I.—The Attitude of the Public.
Institutional Needs ... ... ... ... ... 330
The Public Well-Being ... ... ... ... ... 331
Prison Reform—Scotland's Care for Sanitary Science.
Health Administration by County Councils ... 332
Colonies for Mental Defectives ... ... ... 332
A County Tuberculosis Scheme ... ... ... 332
Parliament and the Professions ... ... ... 333
The Editor's Letter-Box ... ... ... ... 334
The Sites of Mental Hospitals—Diplomas in Radiography.
The Matrons' and Sisters' Department ... ... 335
Nurses Entitled to Registration.
The Scottish Syllabus.
Round the Hospitals ... ... ... ... ... 336
Women's Work in the X-Ray Department ... 338
II.—Qualifications and Study.
Matrons' and Sisters' Appointments ... ... 340
The College of Nursing (Limited by Guarantee) 340

